# Downregulation of vimentin in macrophages infected with live *Mycobacterium tuberculosis* is mediated by Reactive Oxygen Species

**DOI:** 10.1038/srep21526

**Published:** 2016-02-15

**Authors:** P. P. Mahesh, R. J. Retnakumar, Sathish Mundayoor

**Affiliations:** 1 Mycobacteria Research, Bacterial and Parasite Disease Biology, Rajiv Gandhi Centre for Biotechnology, Thycaud P.O., Trivandrum 695014, Kerala, India

## Abstract

*Mycobacterium tuberculosis* persists primarily in macrophages after infection and manipulates the host defence pathways in its favour. 2D gel electrophoresis results showed that vimentin, an intermediate filament protein, is downregulated in macrophages infected with live *Mycobacterium tuberculosis* H37Rv when compared to macrophages infected with heat- killed H37Rv. The downregulation was confirmed by Western blot and quantitative RT-PCR. Besides, the expression of vimentin in avirulent strain, *Mycobacterium tuberculosis* H37Ra- infected macrophages was similar to the expression in heat-killed H37Rv- infected macrophages. Increased expression of vimentin in H_2_O_2_- treated live H37Rv-infected macrophages and decreased expression of vimentin both in NAC and DPI- treated heat-killed H37Rv-infected macrophages showed that vimentin expression is positively regulated by ROS. Ectopic expression of ESAT-6 in macrophages decreased both the level of ROS and the expression of vimentin which implies that *Mycobacterium tuberculosis-*mediated downregulation of vimentin is at least in part due to the downregulation of ROS by the pathogen. Interestingly, the incubation of macrophages with anti-vimentin antibody increased the ROS production and decreased the survival of H37Rv. In addition, we also showed that the pattern of phosphorylation of vimentin in macrophages by PKA/PKC is different from monocytes, emphasizing a role for vimentin phosphorylation in macrophage differentiation.

*Mycobacterium tuberculosis* when phagocytosed by activated macrophages evades the defence strategies of the host and persists indefinitely^1^,^2^. For an effective persistence, virulence determinants of the pathogen probably start acting as soon as it enters the phagocyte. Dampening the inflammatory response of the macrophage is an important strategy used by the pathogenic mycobacteria during the initial period of infection^3^. *M. tuberculosis* is known for downregulating both the production of Reactive Oxygen Species (ROS) and production of pro-inflammatory cytokines by macrophages^4^,^5^.

Interest in the contribution of ROS to pro-inflammatory signalling in macrophages, rather than in the direct microbicidal activity has been growing in recent years^6^,^7^,^8^. ROS may be viewed as a driving force for the sequence of events that happen in activated macrophages leading to the clearance of pathogens^9^,^10^,^11^,^12^. At the same time, too much inflammation is beneficial for the pathogenic bacteria since the resulting necrosis favours the dissemination of bacteria^13^,^14^. Therefore, a sub lethal and balanced inflammation is needed for better containment of pathogens by macrophages.

In our study, we identified that vimentin is downregulated in live *M. tuberculosis*- infected macrophages. Vimentin is an intermediate filament protein^15^. Apart from being a cytoskeletal protein, its alternative roles in cell biology are gaining special interest. Vimentin is associated with macrophage differentiation, phagocytosis and ROS production^16^,^17^. Vimentin is expressed on activated macrophages and is secreted in response to pro-inflammatory stimuli^18^. The cell surface- expressed vimentin of *M. tuberculosis*- infected human monocytes is involved in binding to NKp46 receptor, which may help in the identification of infected monocytes by NK cells^19^. On the other hand, a receptor like property of vimentin is also described^20^,^21^,^22^. Fragmentation of vimentin filaments by caspases was found to promote apoptosis and vimentin was also reported to be associated with inflammatory diseases like arthritis and nephritis^23^,^24^,^25^,^26^,^27^,^28^. At the same time, recent reports show that vimentin is dispensable for inflammation and ROS production^29^,^30^. In this context we looked into the mode of expression of vimentin in stimulated macrophages and propose a mechanism by which vimentin is downregulated by *M. tuberculosis.*

## Results

### Vimentin is downregulated by *Mycobacterium tuberculosis*

**Figure 1:**
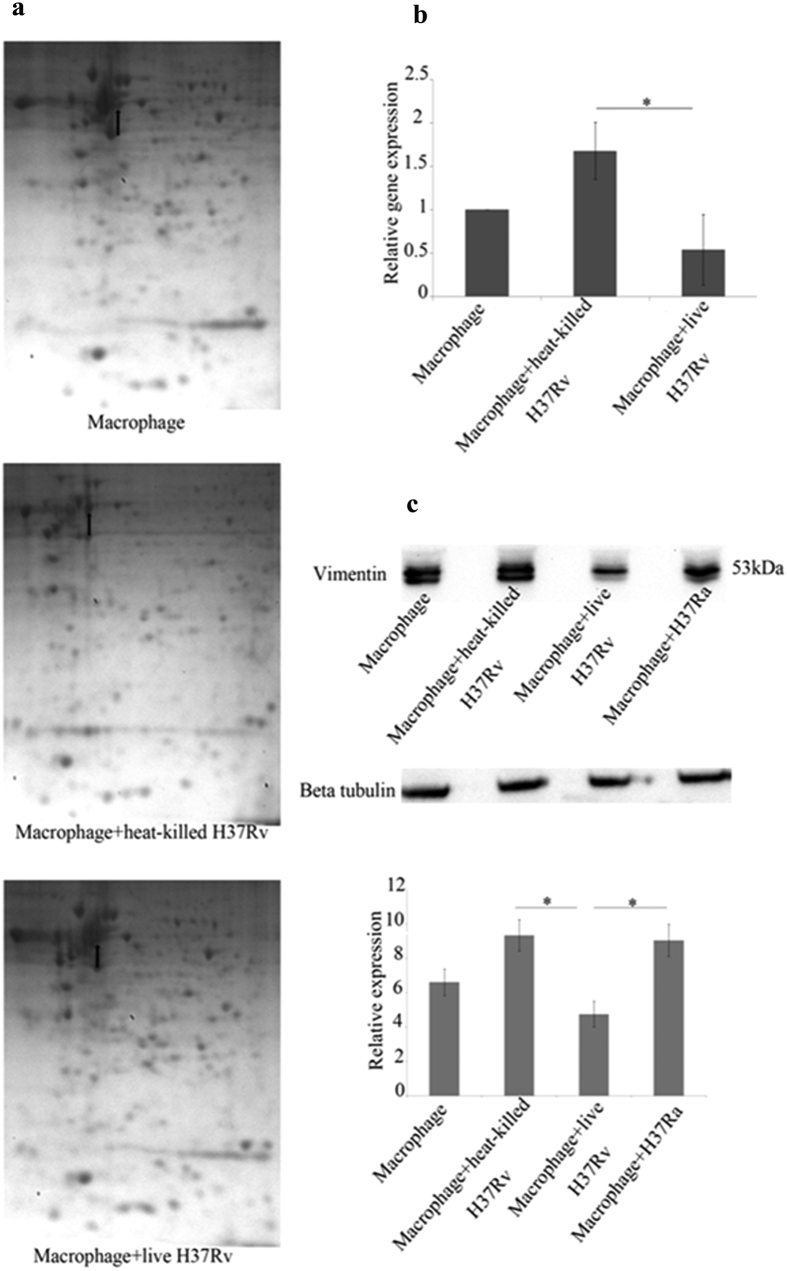
Vimentin is downregulated by live H37Rv. (**a**) Macrophage monolayers were infected with live H37Rv and heat-killed H37Rv and uninfected macrophage was set as control. 2DE was done after 24 h post infection and visualized by Coomassie Blue. The arrow points to the spot picked and later identified as vimentin by MALDI-TOF-TOF. The experiment is a representative of 2 independent experiments with similar results. (**b**) qRT-PCR was done using the RNA isolated from uninfected macrophages and macrophages infected with heat-killed or live *M. tuberculosis* H37Rv, 12 h after infection and setting GAPDH as reference gene. Expression of vimentin in live H37Rv-infected macrophages is decreased when compared to heat-killed H37Rv-infected macrophages or uninfected macrophages. Values are expressed as mean ± SE, n = 3, *Mean difference is significant at 0.05 level. (**c**) Macrophage monolayers were infected with live H37Rv, heat-killed H37Rv and H37Ra. After 12 h of infection different samples were processed for Western blotting. β-tubulin was used as loading control. The expression of vimentin is decreased in live H37Rv-infected macrophages when compared to heat-killed H37Rv infected macrophages, H37Ra-infected macrophages and uninfected macrophages. Values are expressed as mean ± SE, n = 3. *Mean difference is significant at 0.05 level.

THP1- derived macrophages were infected with live or heat killed *Mycobacterium tuberculosis* H37Rv, a virulent laboratory strain, in the multiplicity of infection (MOI) of 20 bacilli/macrophage (a culture of OD = 0.15 at 610 nm contains 3 × 10^8^ bacteria/ml). Total protein of infected macrophages was subjected to 2D gel electrophoresis to screen for proteins that were differentially expressed. Selected proteins were manually picked. One of the differentially expressed proteins was subjected to MALDI-TOF-TOF analysis (Supplementary Fig. S1) and was identified as vimentin. It was found to be upregulated in heat-killed H37Rv- infected macrophages and downregulated in live H37Rv- infected macrophages (Fig. 1a). This observation of ours is in agreement with the microarray data of Spira *et al*.^31^ wherein they showed that vimentin is downregulated in H37Rv- infected alveolar macrophages when compared to H37Ra- infected macrophages. The differential expression of vimentin was further confirmed by quantitative RT-PCR (Fig. 1b) and Western blot analysis (Fig. 1c). We could also find that expression of vimentin in the avirulent strain, H37Ra –infected macrophages was similar to that of heat-killed H37Rv-infected macrophages by Western blot (Fig. 1c). Further, we did Western blot analysis at various time points after infection; 0 h (4 h after addition of bacteria), 2 h, 6 h, 12 h, 24 h and 48 h. The earliest convincing differential expression of vimentin was at 12 h and the expression of vimentin at 24 h and 48 h were similar to that obtained at 12 h (Supplementary Fig. S2). Interestingly, the expression of vimentin was found to show an upregulating tendency both in H37Rv and H37Ra infections when the MOI was 5 bacilli/macrophage (Supplementary Fig. S2), which indicates that there is a need for a threshold number of organisms for eliciting a pathogen specific response. We also confirmed that the viability of H37Rv-infected macrophages after infection at an MOI of 5 bacilli/macrophage and 20 bacilli/macrophage was similar using the MTT assay. Morphology indicated that the macrophages were healthy at 24 h post infection. (Supplementary Fig. S3).

### PKA/PKC phosphorylation of vimentin is a marker for monocyte to macrophage differentiation

**Figure 2:**
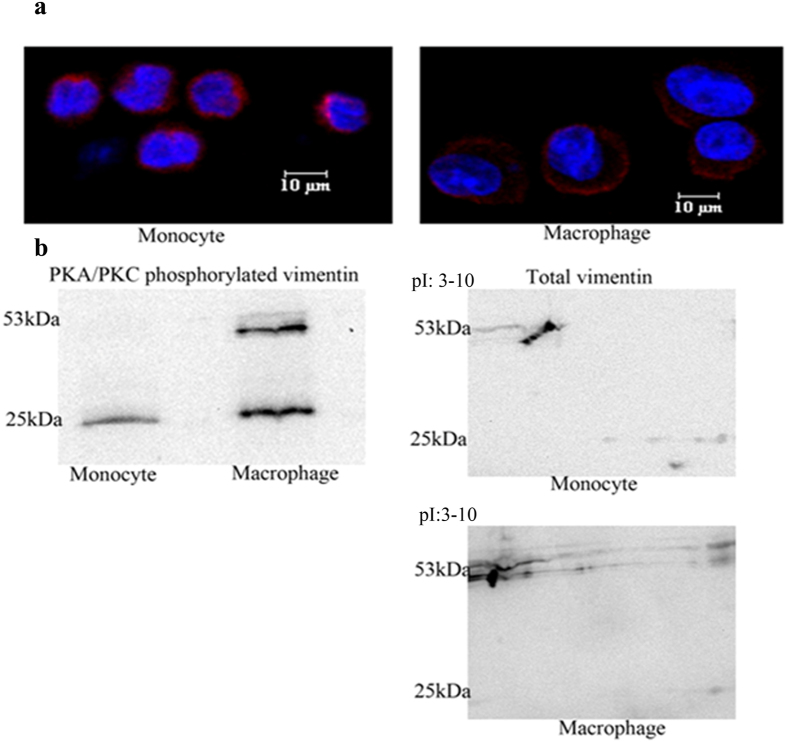
Profile of vimentin in monocytes and macrophages. (**a**) Immunofluorescence of vimentin (red) -stained THP1 monocytes and macrophages clearly demonstrating the difference in the distribution between them (**b**) The left panel shows Western blot of immunoprecipitate from PKA substrate antibody pull down. PKA/PKC phosphorylated molecular size forms of vimentin in monocytes and macrophages demonstrating that phosphorylated proteins around 53 KDa are present only in induced macrophages. The right panel shows Western blots of total vimentin from 2DE experiments showing the presence of both the cluster of spots near to 53 kDa and the 25 kDa form in monocytes as well as in macrophages; the cluster of spots near to 53 kDa in macrophages has shifted left to a more acidic pI compared to monocytes due to phosphorylation.

THP1 monocytic cells were induced to form macrophages by the addition of PMA. We investigated whether this transition induces any change to vimentin, since vimentin exhibits extensive dynamics during cellular processes. The filament disassembly of vimentin, needed for many cellular functions, is influenced by the phosphorylation status of vimentin^32^,^33^,^34^. PKC phosphorylation of vimentin is needed for the periplasmic localization and secretion of vimentin in activated macrophages and cell surface localization is necessary for vimentin to contribute to the inflammatory response of macrophages^18^. We observed that vimentin in THP1 monocytes is evenly distributed in the cytoplasm while it is localized to a greater extent on the cell periphery in macrophages (Fig. 2a). Vimentin exists in different molecular size forms^35^. Our analysis showed that different vimentin molecular size forms are differentially phosphorylated by PKA/PKC in monocytes and macrophages. Total cell lysates of both monocytes and macrophages were immunoprecipitated with a PKA substrate antibody (it also picks up PKC- phosphorylated proteins) and subsequently probed with anti-vimentin antibody by immunoblotting. We observed that a vimentin molecular size form with a size approximately 25 kDa was invariably phosphorylated in both monocytes and macrophages but phoshorylated forms around the size of 53 kDa were present only in macrophages (Fig. 2a). Size forms around 53 kDa could be discerned in monocytes when probed with anti-vimentin antibody but these do not appear to be phosphorylated (Fig. 2b). In conformity with these observations there is another indication of phosphorylation of proteins around 53 kDa size in macrophages in the 2D blot where those protein spots were shifted to the left position, possibly because the phoshorylated protein shifts to a more acidic pI in 2DE compared to its non-phosphorylated form. These observations emphasize a role for phosphorylation of vimentin in the differentiation of monocyte to macrophage and possibly in macrophage activation.

### Downregulation of vimentin in macrophages by live *Mycobacterium tuberculosis* is mediated by ROS

THP1- derived macrophages were infected with live H37Rv and heat-killed H37Rv at an MOI of 20 bacilli/macrophage. Two different sets of treatments were given to macrophages such that i) treatments which were assumed to upregulate vimentin were given to live H37Rv -infected macrophages and ii) treatments which were assumed to downregulate vimentin were given to heat-killed H37Rv- infected macrophages. Therefore, in the first group, live H37Rv -infected macrophages were treated with H_2_O_2,_ and TNFα after 4 h of phagocytosis. TNFα was used as it is a well known pro-inflammatory cytokine known to be important in the host immunity against tuberculosis and is an inducer of ROS^36^. Macrophages were also treated with *E.coli* LPS which is pro-inflammatory and is another inducer of ROS^37^. In the second group, heat-killed H37Rv- infected macrophages were treated with N-acetyl L-cysteine (NAC), a scavenger of ROS; pyrrolidine dithiocarbamate (PDTC), an inhibitor of NF-ĸB; GÖ6983, an inhibitor of PKC and diphenylene iodonium chloride (DPI). It has been reported that cell surface expression of vimentin in macrophages is enhanced by PKC phosphorylation^18^ and PKC is also an activator of NADPH oxidase. DPI is an inhibitor of both NADPH oxidase and nitric oxide synthase. All the samples were processed for Western blot after 12 h post infection.

**Figure 3:**
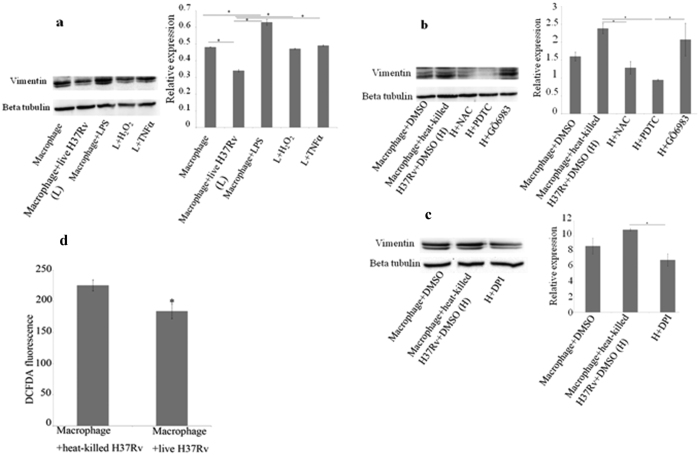
Downregulation of vimentin by *Mycobacterium tuberculosis* relates to the pathogen’s anti-inflammatory effect on macrophages. (**a**) Macrophage monolayers were infected with live H37Rv. Infected macrophages were treated with H_2_O_2_ at100 μM and TNFα at10 ng/ml, after 4 h of phagocytosis. Uniinfected macrophages were treated with LPS at1 μg/ml. After 12 h post infection, all the samples were processed for Western blotting and probed with anti-vimentin antibody; β-tubulin was used as loading control. Live mycobacteria reduce vimentin expression and this is restored by H_2_O_2_ and TNFα. LPS increases vimentin and is used as a positive control. Values are expressed as mean ± SE, n = 3, *Mean difference is significant at 0.05 level. (**b,c**) Macrophage monolayers were infected with heat-killed H37Rv. Infected macrophages were treated with NAC at10 mM, PDTC at 50 μM, GÖ6983 at 500 nM and DPI at10 μM, after 4 h of phagocytosis. After 12 h post infection, all the samples were processed for Western blotting and probed with anti-vimentin antibody; β-tubulin was used as loading control. Values are expressed as mean ± SE, n = 3, *Mean difference is significant at 0.05 level. (**d**) The level of ROS is found to be decreased in live H37Rv-infected macrophages compared to heat-killed H37Rv-infected macrophages after 24 h of infection. Values are expressed as mean ± SE, n = 4 and p = 0.03.

LPS-treated macrophages showed increased expression of vimentin compared to untreated macrophages. When live H37Rv-infected macrophages were treated with TNFα or H_2_O_2,_ the expression of vimentin is brought back to almost the same level as in uninfected macrophages (Fig. 3a), which shows that vimentin expression is upregulated by both of the stimuli. NAC, PDTC and DPI-treated heat-killed H37Rv-infected macrophages showed a large decrease in the expression of vimentin, while GÖ6983 treated heat-killed H37Rv-infected macrophages did not show a significant decrease in the expression when compared to heat-killed H37Rv- infected macrophages (Fig. 3b,c). Thus, inhibiting NF-ĸB (by PDTC) or scavenging ROS (by NAC) reverses the increase in vimentin expression caused by heat-killed mycobacteria. Similar reversal was also obtained on treatment of heat-killed H37Rv-infected macrophages by DPI which is an inhibitor of both NADPH oxidase and nitric oxide synthase. All these observations show that ROS plays an important part in the expression of vimentin.

Taken together, the increased expression of vimentin in H_2_O_2_- treated live H37Rv-infected macrophages and decreased expression of vimentin both in NAC and DPI- treated heat-killed H37Rv-infected macrophages suggest that vimentin expression is positively regulated by ROS. Had ROS been dispensable in the increase in expression of vimentin in heat-killed bacteria stimulated macrophages, then the ROS dependent pathway could have been bypassed to retain the increase in expression of vimentin when the macrophages were treated with NAC or DPI. We could also show that the level of ROS in live H37Rv-infected macrophages is significantly lower than that in heat-killed H37Rv-infected macrophages by measuring the fluorescence of DCFDA (Fig. 3d). When the observations that the upregulation of vimentin by heat-killed H37Rv and downregulation of vimentin by live H37Rv are considered together with the results from different treatments in the two groups as mentioned above, and the level of ROS measured in live H37Rv-infected macrophages and heat-killed H37Rv-infected macrophages is taken into account, it is reasonable to assume that downregulation of vimentin by live *M. tuberculosis* is at least partially due to the downregulation of ROS by the pathogen. The finding of Helguera-repetto *et al*. that the level of ROS decreased in *M. tuberculosis-*infected macrophages is in support of this assumption^4^. Vimentin is a known NF-ĸB target gene and inhibition of NF-ĸB reduces the expression of vimentin^38^. Our observation that the expression of vimentin is decreased in PDTC (an inhibitor of NF-ĸB and also an anti-oxidant)-treated heat-killed H37Rv-infected macrophages validates the earlier finding. Upregulation of vimentin by LPS and TNFα (both of them induce ROS production) shows that vimentin is a target of pro-inflammatory pathways of activated macrophages.

### Ectopic expression of ESAT-6 in macrophages downregulates both the level of ROS and the expression of vimentin

**Figure 4:**
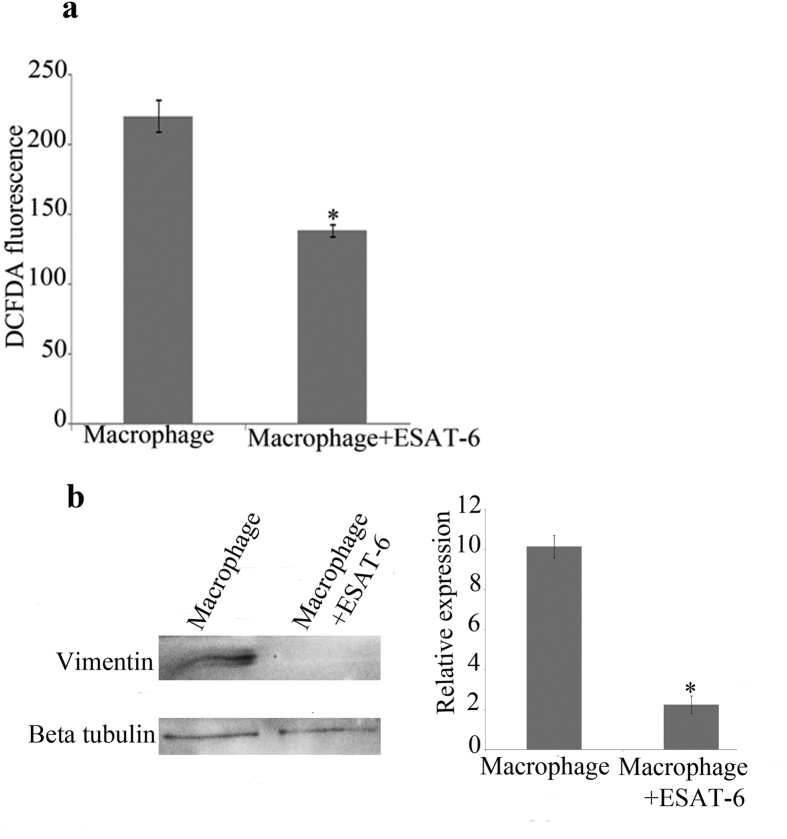
ESAT-6 downregulates both the level of ROS and the expression of vimentin. THP1 cells were transfected with either plasmid containing ESAT-6 or the control plasmid and treated with PMA to form macrophages. (**a**) The level of ROS measured as DCFDA fluorescence is decreased in ESAT-6 expressing macrophages compared to the control. Values are expressed as mean ± SE, n = 9. *Mean difference is significant at 0.05 level. (**b**) Expression of vimentin is decreased in ESAT-6 expressing macrophages compared to the control. Values are expressed as mean ± SE, n = 3. *Mean difference is significant at 0.05 level.

Since the downregulation of expression of vimentin is probably through the secretion of a mycobacterial product by live mycobacteria, we decided to investigate the role of such products. Earlier studies have shown that secreted proteins such as ESAT-6 have roles in virulence and pathogenicity^39^. ESAT-6 is one of the abundantly secreted proteins of pathogenic mycobacteria. Therefore we wanted to investigate the effect of intracellular expression of ESAT- 6 in macrophages on the expression of vimentin and production of ROS. ESAT-6 was cloned into pIRES with ECFP as reporter and transfected into THP1 monocytes as described in methods. After 24 hours, viable cells were counted, seeded into culture plates and treated with PMA. Following 24 hours of PMA treatment, the differentiated macrophages were processed for ROS measurement and Western blot analysis. Macrophages transfected with the plasmid control was used for comparison. We could find that both the level of ROS (Fig.4a), as measured by DCFDA fluorescence and the expression of vimentin (Fig.4b) were decreased significantly in ESAT-6-transfected macrophages compared to the controls. Earlier, Ganguly, N. *et al*.^40^ reported that incubation of macrophages with purified ESAT-6 and CFP-10 or their fusion product downregulated ROS and here we confirm their observation using macrophages expressing ESAT-6 internally. In the present study we have been able to show that vimentin has a ROS dependent expression in *M. tuberculosis*-infected macrophages. Correlating the downregulation of vimentin expression to the decreased level of ROS in ESAT-6 expressing macrophages allows us to assume that *M. tuberculosis-*mediated downregulation of vimentin is at least in part due to the downregulation of ROS by the pathogen. Further, the similarity in expression of vimentin in heat-killed H37Rv infection and H37Ra infection is possibly due to the absence of ESAT-6 in both conditions; via heat-killing in the former and an attenuating mutation in the PhoP regulator of H37Ra which controls the secretion of ESAT-6 in the latter^41^ such that there is no secretion of ESAT-6.

### Treatment of macrophages with anti-vimentin antibody increases ROS production and killing of *M. tuberculosis*.

**Figure 5:**
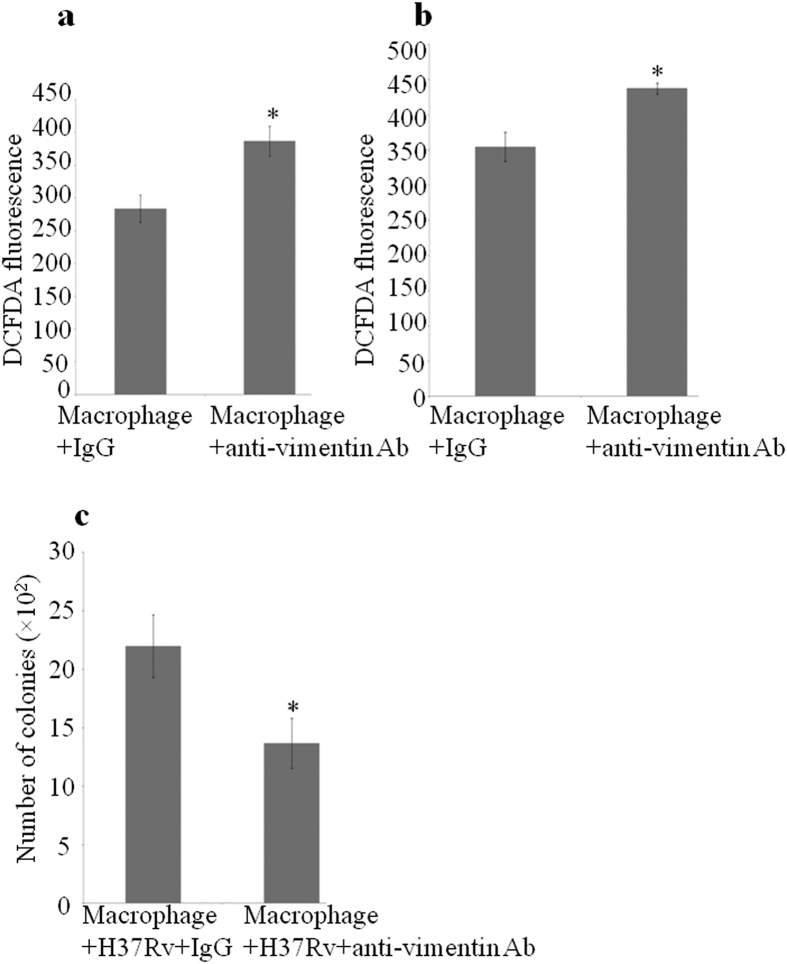
Treatment of macrophages with anti-vimentin antibody increases ROS level and killing of *M. tuberculosis.* Uninfected macrophage monolayers were treated with anti-vimentin antibody or normal IgG and ROS was measured as DCFDA fluorescence. (**a**) at 6 h; n = 8, p = 0.005 and (**b**) at 24 h; n = 7, p = 0.004. (**c**) Macrophage monolayers were infected with H37Rv and treated with anti-vimentin antibody or normal IgG after phagocytosis. After 24 h post infection, the cells were lysed, diluted to 10^−2^ and plated; n = 8, p = 0.034. Values are expressed as mean ± SE. CFU measurements show that anti-vimentin antibody treatment decreases the number of live H37Rv.

The cell surface-expressed vimentin was earlier reported to have a role in ROS production in activated macrophages^18^. We wanted to check the same in our case. For that, uninfected macrophages were treated with anti-vimentin antibody for 6 h and 24 h. Intracellular ROS was measured as DCFDA fluorescence. ROS level in anti-vimentin antibody- treated macrophages was more than that in normal IgG- treated control macrophages at both time points (Fig. 5a,b). We designed the experiment of anti-vimentin antibody treatment to macrophages with a pre-conception that it would neutralize the surface- expressed vimentin. But we got entirely the opposite result that anti-vimentin antibody- treated macrophages gave higher ROS levels than the isotype controls at both 6 h and 24 h in all replicates. This deviation could be possibly explained by suggesting that instead of neutralizing surface-expressed vimentin, the antibody binding might have resulted in its activation. This may be due to the fact that the surface- localized vimentin has receptor like properties as they have clearly defined head, tail and rod domains^27^ and the antibody might be acting as a ligand for the exposed receptor domain and activating the same. The immunogen of the antibody we used (ab16700, abcam) is the full length recombinant 53 kDa protein which may cause an exact binding of antibody to the exposed domains of vimentin. We find that studies on the binding of autoantibodies to the surface- expressed proteins in inflammatory diseases has a similarity with our observation^26^,^28^ and it is known that in Graves’ disease the autoantibody to TSH receptor mimics TSH which is the ligand for the receptor and the antibody binding activates the receptor^42^. Further, when H37Rv- infected macrophages were treated with anti-vimentin antibody, a significant increase in the killing of bacteria was found when compared to the normal IgG- treated control (Fig. 5c). Interestingly, a recent study by Nirit Mor-Vaknin *et al*.^30^ show that in murine colitis, vimentin knockout mice were able to kill *E. coli* better, contrary to their earlier finding.

### CORD analysis predicts that vimentin is co-expressed with a set of pro-inflammatory mediators

Even though vimentin is reported to be associated with a variety of inflammatory conditions its exact function is not clear. It can bind to IgG and activate the complement cascade^43^,^44^. To identify the probable co-regulated genes for vimentin we made use of CORD analysis which is a web-enabled program to determine gene-gene correlations across different gene expression microarray platforms^45^. According to the authors, the analysis helps an investigator to explore and generate novel hypotheses from data available in the public data repositories and the list of co-expressed genes may provide valuable information about previously unappreciated biological connections for known genes. We could identify several pro-inflammatory mediators from the “concordant” set of genes (Table 1) given by the analysis which gives some indications about the possible pathways where vimentin has a functional role. This data helps us to speculate that vimentin is expressed as a part of the inflammatory response of respective cells. The complete file generated by the CORD analysis is given as supplementary information.

## Discussion

Vimentin, an intermediate filament protein is widely described as a marker of epithelial-mesenchymal transition. Our work underscores the contribution of vimentin in the differentiation of monocytes to macrophages. We showed that vimentin molecular size forms are differentially phosphorylated by PKA/PKC in monocytes and macrophages. Molecular size forms near to 53 kDa are only phosphorylated in macrophages and this may be linked to vimentin filament disassembly followed by cell surface localization.

*Mycobacterium tuberculosis* is a highly adapted pathogen probably due to its co-evolution with humans^46^. It employs various strategies for establishing itself as a persisting pathogen. As macrophages are its primary host cells, it subverts many pathways of activated macrophages. Activated macrophage generally undergoes a well programmed sequence of events when it encounters a pathogen, which includes phagocytosis, ROS production, trafficking of pathogen- laden phagosomes, phagosome maturation, fusion of phagosomes with lysosomes and antigen presentation. Besides, it may undergo apoptosis, autophagy and necrosis under different situations. The exact sequence of events that take place and mechanisms that channel these events are being unravelled now. In the present study, we show that live *M. tuberculosis* downregulates vimentin while the avirulent and heat killed forms upregulate the same. In addition, the downregulation requires a threshold number of organisms that are metabolically active. In this study, we propose a mechanism for the downregulation of vimentin in live *M. tuberculosis-*infected THP1- derived macrophages. Our results present a picture of a ROS dependent expression of vimentin in macrophages and also underline the observation that the expression of vimentin is positively influenced by pro-inflammatory stimuli. We also show that the treatment of macrophages with anti-vimentin antibody resulted in increased ROS production and killing of the pathogen.

Secretory proteins of *M. tuberculosis* such as ESAT-6 and CFP-10 were found to inhibit LPS- induced NF-ĸB transactivation by downregulation of ROS production^40^ and *M. tuberculosis* Sigma factor E regulon was found to modulate the host inflammatory response^47^. We could also show that ectopic expression of ESAT-6 in macrophages downregulates both the level of ROS and the expression of vimentin indicating that downregulation of vimentin by *M. tuberculosis* is at least in part via decreasing the level of ROS. *M. tuberculosis* is also known to bypass NF-ĸB signalling^48^. This can be supplemented with another observation that patients with chronic granulomatous disease who are genetically deficient in NOX2 have increased susceptibility to infection with non-pathogenic vaccine strain, BCG^49^. Further, by CORD analysis we suggest that vimentin is co-expressed with a set of pro-inflammatory mediators.

Since we have shown that vimentin has a ROS dependent expression and vimentin expression is downregulated in PDTC-treated heat-killed H37Rv-infected macrophages, it may be speculated that vimentin expression in stimulated macrophages is positively affected by ROS-mediated NF-ĸB activation^50^. Further, the level of ROS along with intracellular calcium in a stimulated cell was shown to have control over the ultimate fate of downstream pathways^51^. Our data show that live virulent *M. tuberculosis* causes a significant reduction in the total ROS level possibly via ESAT-6 and/ or similar proteins in the infected macrophages when compared to heat-killed bacterial infection. This suggests that the downregulation of ROS is likely to be an important strategy employed by the pathogen for its persistence during the early period of infection. Since there are many ESAT-6 like proteins and there are duplications of the genomic region containing ESAT-6 gene in *M. tuberculosis*, a concerted action of proteins with similar function is likely to take place. *M. tuberculosis* is known to cause a phenotypic switching from M1 polarized macrophages (pro-inflammatory) to M2 polarized macrophages (anti-inflammatory) in granulomas and NF-ĸB signalling plays a critical role in the containment of pathogen during the early period of infection^52^. Besides, the loss of NADPH oxidase- derived ROS was reported to promote M2 phenotype in macrophages^53^ and NADPH oxidase deficiency was shown to cause increased polymorphonuclear leukocyte infiltration following *E.coli* challenge^54^, and both these findings are applicable to tuberculous granulomas.

In conclusion, we have shown that vimentin expression in macrophages is upregulated by pro-inflammatory stimuli like TNFα, LPS and H_2_O_2_ while it is downregulated by NAC, DPI and PDTC. These observations suggest that the expression of vimentin is a part of an inflammatory response of macrophages that is optimally needed for the containment of pathogens and the downregulation of vimentin by *M. tuberculosis* may be viewed as an effect of the negative modulation of the inflammatory response of macrophages, possibly in a ROS-dependent manner. These effects seem to be controlled through secreted components of the bacteria which subtly subvert macrophage functions. Finally, from our study and those of others, we propose that downregulation of ROS by *M. tuberculosis* as a fundamental mechanism to create an anti-inflammatory milieu in activated macrophages which aids the pathogen to persist intracellularly.

## Methods

### Cell culture, bacteria and infection

THP1 monocytic cell line was maintained in RPMI-1640 medium (R4130, Sigma) supplemented with 10% FBS. For obtaining macrophage monolayer THP1 cells with required cell density (3 × 10^6^ cells in T-25 flask and 4 × 10^4^ cells per well in a 96 well plate) were treated with 20 ng/ml of PMA (P8139, Sigma) and after one day PMA was washed off 2 times with RPMI. After washing the cells 3ml and 100 μl complete medium was maintained until the addition of bacteria in T-25 flask and 96 well plate respectively (for 96 well plate bacteria were added in a 100 μl suspension). Stocks of *Mycobacterium tuberculosis* strains, H37Rv and H37Ra were prepared using beads (PROTECT bacterial preservers) and stored at −80 °C. Periodically, the frozen stocks were revived in7H9 broth and streaked on LJ slants and maintained for at least 2 months in an incubator at 37 °C. Prior to experiments bacteria were inoculated into 7H9 broth (271310, Difco) from LJ slants. 7–10 days old broth cultures were used for infection experiments. An OD of 0.15 at 610 nm was measured to give McFarland standard 1 and was calculated to contain 3 × 10^8^ bacteria/ml. On the day of infection log phase culture of bacteria was collected and passed through a syringe for 20 times and kept for 10 min to remove the clumps and the required volume of the culture was pelleted by centrifugation at 3000 g for 10 min. Then the pellet was resuspended in RPMI and added to the macrophage monolayer. For increasing the infection efficiency, minimum volume of bacterial suspension was used to infect monolayer of macrophages. After the addition of bacteria the macrophage monolayer was kept for 4 h for phagocytosis. After 4 h the extracellular bacteria were washed off 3 times with RPMI and complete medium was added.

### Chemicals

Chemicals and their respective concentrations used for the treatment of infected and uninfected macrophages are listed below. H_2_O_2_^−^100 μM, NAC (A7250, Sigma) −10 mM, PDTC (P8765, Sigma) −50 μM, GÖ6983 (G1918, Sigma) −500 nM, DPI (D2926, Sigma) −10 μM, LPS (L4391, Sigma) −1 μg/ml and recombinant TNFα (RTNFAI, Thermo) −10 ng/ml.

### 2D gel electrophoresis

After 24 h of infection, total protein of infected and uninfected macrophages was isolated using 2D lysis buffer composed of 8M urea, 1M thiourea and 4% CHAPS along with 1 mM PMSF. After 30 minutes of incubation at room temperature the cell lysate was centrifuged at 14000 g for 20 min at 18 °C and then 300 μg of total protein was loaded on a 7cm IPG strip of 3–10 pI range (163–2000, BIO RAD). After isoelectric focusing, the strips were equilibrated and PAGE (15% gel) was run to resolve the proteins. From the gel, visually identified spot was picked and analyzed by MALDI-TOF-TOF.

### Quantitative RT-PCR

After 12 h of infection, total RNA of infected and uninfected macrophages was isolated using Illustra RNAspin (25-0500-71, GE) and the concentration was estimated by NanoVue Plus, GE. Primers for the genes were designed by Primer Premier Software. Primer pair for vimentin: FP-5′ TGAACGCAAAGTGGAA 3′, RP-5′ AGGTCAGGCTTGGAAA 3′ and primer pair for the reference gene GAPDH: FP: 5′ TCAAGAAGGTGGTGAAGCA 3′, RP: 5′ AGGTGGAGGAGTGGGTGT 3′. Primer efficiency for vimentin was 90.1% and that of GAPDH was 92.15%. qRT-PCR was performed using iScript one step RT-PCR kit (170-8892, BIO RAD). The relative quantitation and calculation of primer efficiency were done by the same software (CFX manager, BIO RAD) which performed the RT-PCR.

### Western blot and immunoprecipitation

At various time points, infected and uninfected macrophage monolayers were washed with ice cold PBS, scraped in 1ml PBS and were centrifuged at 2000 rpm for 5 min at 4 °C. The cell pellet was lysed with RIPA buffer (R0278, Sigma) in the presence of protease inhibitor cocktail (P8340, Sigma). After 5 min of incubation on ice, the cell lysate was centrifuged at 14000 g for 10 min at 4 °C and supernatant was collected. Protein was estimated using a BCA kit (23225, Thermo) and loaded at 15 μg per well. β-tubulin (ab6046, abcam) was used as loading control. Blot was probed by anti-vimentin antibody (ab16700, abcam) and subsequently with anti-rabbit secondary antibody HRP (sc-2004, Santa Cruz), and was developed using ECL Prime (RPN2232, GE). Relative expression was calculated by taking the ratio of intensities of vimentin to β-tubulin using Quantity One software (BIO RAD).

For immunoprecipitation, total protein of monocytes and macrophages was isolated using the lysis buffer composed of 20 mM Tris (pH: 7.5), 150 mM NaCl and 1% Igepal CA-630 (I8896, Sigma) in the presence of protease inhibitor cocktail and phosphatase inhibitor cocktail 2 (P5726, Sigma) & cocktail 3 (P0044, Sigma). After keeping 10 min on ice the cell lysate was centrifuged at 14000 g for 10 min at 4 °C. To 200 μl of the supernatant, 4 μl of PKA substrate antibody (9621, Cell Signaling) was added and incubated at 4 °C overnight on a rocker. After the incubation a 40  μl of 50% protein A agarose slurry (9863S, Cell Signaling) was added and incubated at 4 °C for 3 h on a rocker. The immunoprecipitate was washed 5 times with lysis buffer, loaded and PAGE was run. After that, Western blot was performed and probed with vimentin antibody. Since the antibodies used for pull down and probing were rabbit IgG and vimentin’s MW is 53 kDa which is close to the MW of antibody heavy chain, a conformation specific secondary antibody conjugated with HRP (5127S, Cell Signaling) was used. Blot was developed using ECL method.

### Immunofluorescence

THP1 cells were seeded in an optical bottom 96 well plate at 4 × 10^4^ cells per well and induced to form macrophage monolayer. The cells were fixed by 4% paraformaldehyde, permeabilized by 0.1% Triton X100 and blocked with 3% BSA and 0.3M glycine in PBS. Anti-vimentin antibody was added at 1:200 dilution in PBS with 3% BSA and the secondary antibody used was goat anti-rabbit F(ab’)_2_ conjugated with DyLight 550 (ab102292, abcam). Images were taken under 40X objective with a confocal microscope (Nikon). For obtaining the images of monocytes, all the steps of the immunofluorescence were done in a 1.7ml tube and finally transferred to the plate.

### Antibody treatment, measurement of ROS and plating

For ROS measurement macrophage monolayers were treated with 1:100 dilution of vimentin antibody and 2 μg/ml of normal rabbit IgG (sc-2027, Santa Cruz) was used as control. After 6 h and 24 h the uninfected macrophage monolayers were washed 3 times with RPMI medium. Then 10 μM DCFDA (D6883, Sigma) containing serum free medium (RPMI alone) was added and incubated for 30 min, washed 2 times with PBS and fluorescence was measured using the device Infinite 200 with the application Tecan i-control by setting excitation wave length, 485 nm and emission wave length, 520 nm. To find out the effect of anti-vimentin antibody treatment on the survival of bacteria, macrophages were infected with H37Rv. After 4 h of phagocytosis gentamicin was added to the medium at 15 μg/ml and incubated for 1 h to kill the extracellular bacteria, then washed 3 times with RPMI. After 24 h the cells were lysed with sterile distilled water, diluted to 10^−2^ and plated on 7H10 agar (262710, Difco) supplemented with 0.1% casitone (0259-17, Difco). Colonies were counted after 3 weeks.

### Ectopic expression of ESAT-6 in macrophages

ESAT-6 was amplified from H37Rv DNA using the following primers; FP: 5′ CGGAATTCCGATGACAGAGCAGCAGTGGAAT3′ with EcoR1 site and RP: 5′CGACGCGTCGCTATGCGAACATCCCAGTGACG3′ with Mlu1 site. The amplified fragment was cloned into MCS A of pIRES (Clontech). The reporter gene ECFP was taken from pECFP-N1 (Clontech) by restriction digestion at Sal1 and Not1 sites and ligated into MCS B of pIRES. The insertions were confirmed by both restriction digestion and sequencing. The plasmid was isolated using an endotoxin free plasmid isolation kit (EndoFree Plasmid Maxi Kit, Cat. No. 12362, QIAGEN). Before transfection THP1 cells were washed in serum free RPMI for 2 times. Then a 40 μg of plasmid was mixed with 40 × 10^6^ THP1 monocytes suspended in serum free RPMI in a 4 mm electroporation cuvette and electroporated at the given conditions; choose mode-LV, pulse length-5 ms, charging voltage-350 V in a BTX Harvard Apparatus, ECM 830. Cells were incubated for 10 min on ice before and after electroporation and transferred to culture flasks containing RPMI + 10%FBS and incubated for 24 hrs. After 24 hrs the viable cells were counted using Trypan blue (T8154, Sigma) staining, seeded into plates at respective densities and treated with PMA for next 24 hrs.For Western blot a 10 μg of total protein was loaded. Transfection of cells was confirmed by imaging the cells with 405 nm laser for ECFP expression with a confocal microscope (Supplimentary Fig. S5).

### CORD analysis

Co-regulated genes for vimentin were obtained by submitting required information in the web page http://cord-db.org/. The settings provided for the analysis were, Species = human, Minimum log2 fold change = 1, Minimum p- value = 0.05, Minimum samples per experiment = 2.

### Statistical analysis

Statistical analysis was done using IBM SPSS statistics software, version 19. Comparison of means was done by one way ANOVA (Tukey’s method) by setting alpha value as 0.05. Values were expressed as mean ± SE.

## Additional Information

**How to cite this article**: Mahesh, P. P. *et al*. Downregulation of vimentin in macrophages infected with live *Mycobacterium tuberculosis* is mediated by Reactive Oxygen Species. *Sci. Rep.* **6**, 21526; doi: 10.1038/srep21526 (2016).

## Supplementary Material

Supplementary Information

Supplementary Dataset 1

## Figures and Tables

**Table 1:** Selected co-regulated genes for vimentin.

Co-regulated gene	Description (adopted from NCBI Gene)	Number of experiments	Percentage of concordance	Pearson coefficient	Pearson p-value
Lectin, galactoside- binding, soluble, 1 (LGALS1)	Implicated in cell-cell and cell-matrix interactions, also pro-apoptotic.	1852	88.012	0.595	5.128e-298
Reticulocalbin 1, EF-hand calcium binding domain (RCN1)	Reticulocalbin 1 is a calcium-binding protein located in the lumen of the ER	1413	80.537	0.583	1.130e-282
Interferon induced transmembrane protein 3 (IFITM3)	It acts as an effector molecule for interferon-gamma, which is essential for anti-tuberculosis immune response	1354	86.336	0.574	7.978e-272
Annexin A5 (ANXA5)	Binds cooperatively to phosphatidylserine on apoptotic cell membranes	1473	92.328	0.570	4.417e-268
Transforming growth factor, beta receptor II (TGFBR2)	A receptor with Ser/Thr kinase activity	1478	85.250	0.549	2.105e-244
Notch 2 (NOTCH2)	Regulates interactions between physically adjacent cells.	1402	84.094	0.539	5.106e-234
G protein-coupled receptor 124 (GPR124)	A member of GPCR family	1178	85.653	0.525	7.926e-220
Kruppel-like factor 2 (lung) (KLF2)	Regulates T-cell trafficking by promoting expression of S1P1 and the CD62L.	1418	81.734	0.516	4.316e-211
Platelet-derived growth factor receptor, beta polypeptide (PDGFRB)	A cell surface tyrosine kinase receptor	1509	82.571	0.486	3.984e-184
Transforming growth factor, beta receptor III (TGFBR3)	A membrane proteoglycan that often functions as a co-receptor with other TGF-beta receptors.	1551	80.141	0.453	1.323e-157
Mannose receptor, C type 2 (MRC2)	Important in extracellular matrix remodelling and in phagocytosis	1417	81.016	0.453	4.953e-157
Prostaglandin I2 (prostacyclin) synthase (PTGIS)	Associated with inflammation in atherosclerosis	1500	77.466	0.446	7.145e-152
Chemokine (C-C motif) ligand 2 (CCL2)	This cytokine displays chemotactic activity for monocytes and basophils	1357	78.555	0.430	3.820e-140
Tumor necrosis factor receptor superfamily, member 1A (TNFRSF1A)	This receptor can activate NF-kappaB, mediate apoptosis, and function as a regulator of inflammation.	1131	79.929	0.389	3.161e-113
Fc fragment of IgG, receptor, transporter, alpha (FCGRT)	A receptor that binds the Fc region of monomeric immunoglobulin G.	1416	73.658	0.384	4.921e-110
Protein kinase C, delta binding protein (PRKCDBP)	A binding protein of the protein kinase C, delta	1256	78.662	0.382	1.718e-108
Interleukin 6 (IL6)	Causes acute and chronic inflammation	1079	84.059	0.369	3.657e-101
Complement component 1, s subcomponent (C1S)	A serine protease and constituent of the human complement subcomponent C1	1677	75.074	0.366	2.447e-99
Nuclear factor of kappa light polypeptide gene enhancer in B-cells inhibitor, alpha (NFKBIA)	A member of the NF-kappa-B inhibitor family, which contains multiple ankrin repeat domains.	1314	76.331	0.356	1.192e-93
Complement component 1, r subcomponent (C1R)	The first protease activated in the classical complement pathway	1323	78.155	0.355	3.442e-93
Tumor necrosis factor, alpha-induced protein 6 (TNFAIP6)	This gene can be induced by tumor necrosis factor alpha and interleukin-1	1153	78.577	0.349	7.049e-90
Calumenin (CALU)	A calcium-binding protein localized in the endoplasmic reticulum	1166	80.102	0.348	1.922e-89

CORD analysis was done to find out probable co-regulated genes for vimentin from different micro-array experiments. A few selected co-expressed genes which are important in pro-inflammatory signalling are listed. Detailed data is given as supplementary material.
