# Supplementary material for: Downregulation of vimentin in macrophages infected with live Mycobacterium tuberculosis is mediated by Reactive Oxygen Species
Source: Sci Rep. 2016 Feb 15;6:21526. doi: 10.1038/srep21526 (PMC4753491; doi:10.1038/srep21526)
Supplement: Supplementary Information [file srep21526-s1.pdf]

Title: Downregulation of vimentin in macrophages infected with live *Mycobacterium tuberculosis* is mediated by Reactive Oxygen Species

Authors: Mahesh P.P, Retnakumar R. J and Sathish Mundayoor*

Author addresses and affiliation:

Mycobacteria Research, Bacterial and Parasite Disease Biology, Rajiv Gandhi Centre for Biotechnology, Thycaud P.O., Trivandrum 695014, Kerala, India.

Email: mahesh@rgcb.res.in, retnakumarrj@rgcb.res.in, *smundayoor@rgcb.res.in

a

b

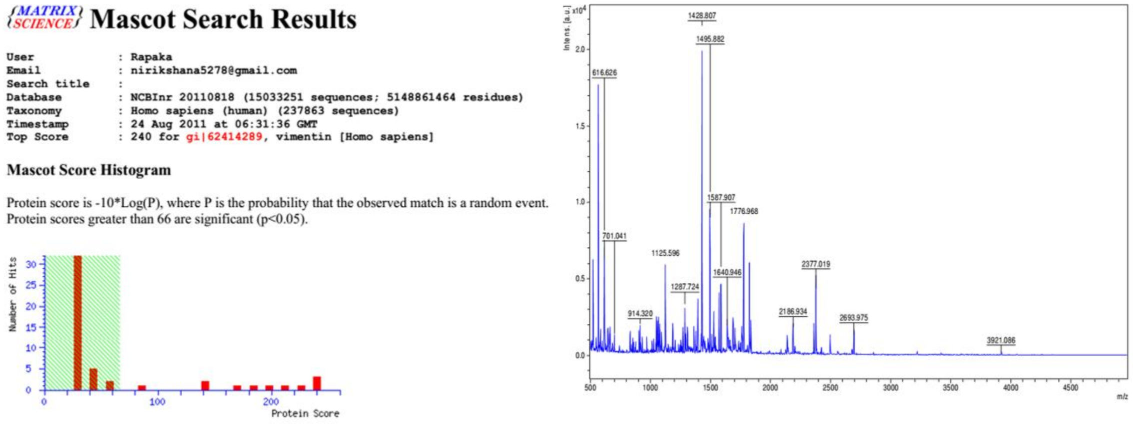

Supplementary figure S1. Identification of vimentin by MALDI-TOF-TOF. The spot picked from 2D gel was analysed by MALDI-TOF-TOF and identified as vimentin. a) Mascot search result for vimentin, b) Mass spectrum for vimentin.

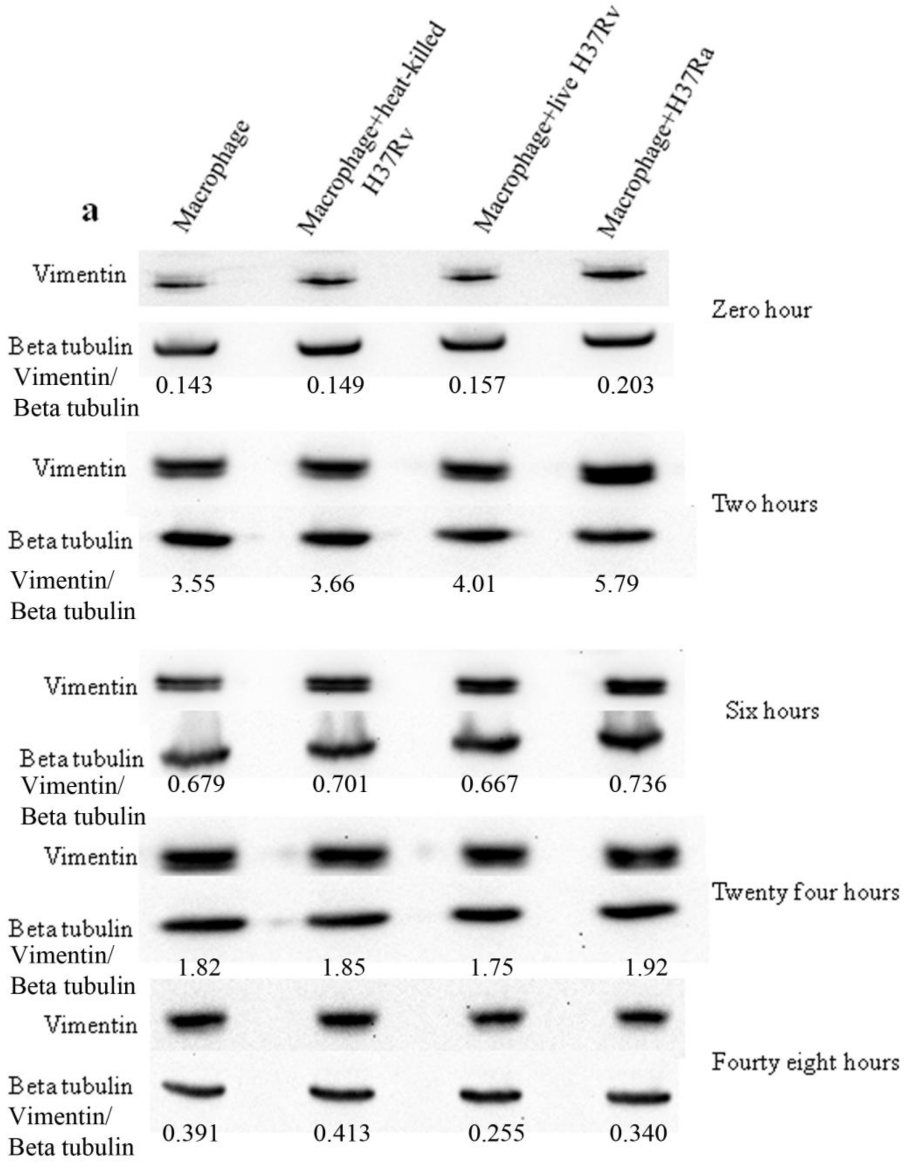
a
Macrophage
Macrophage+heat-killed H37Rv
Macrophage+live H37Rv
Macrophage+H37Ra
Vimentin
Beta tubulin
Vimentin/ Beta tubulin
0.143 0.149 0.157 0.203
Zero hour
Vimentin
Beta tubulin
Vimentin/ Beta tubulin
3.55 3.66 4.01 5.79
Two hours
Vimentin
Beta tubulin
Vimentin/ Beta tubulin
0.679 0.701 0.667 0.736
Six hours
Vimentin
Beta tubulin
Vimentin/ Beta tubulin
1.82 1.85 1.75 1.92
Twenty four hours
Vimentin
Beta tubulin
Vimentin/ Beta tubulin
0.391 0.413 0.255 0.340
Fourty eight hours

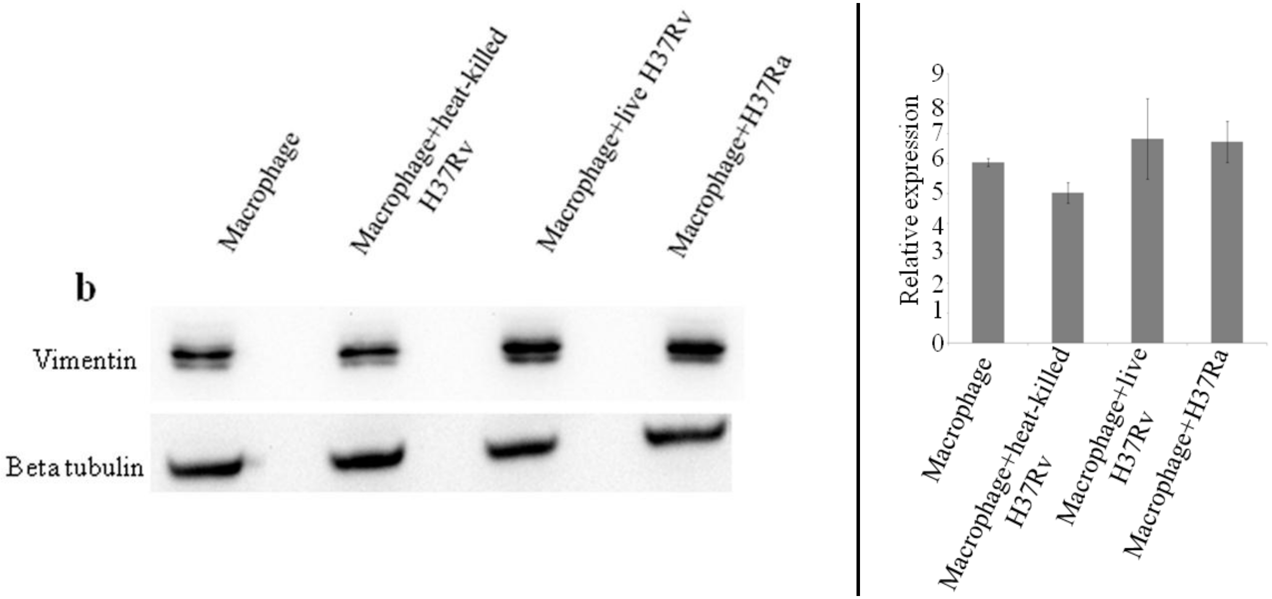

Supplementary figure S2. a) Western blots of vimentin at various time points after infection. 4h after phagocytosis was taken as zero hour. Beta tubulin was used as loading control. Values at the bottom of each lane gives the ratio of vimentin to beta-tubulin b) Expression of vimentin in different infections of macrophages at 12h and at a MOI of 5 bacilli/macrophage. Vimentin expression shows an upregulating tendency both in live virulent H37Rv-infected macrophages and avirulent H37Ra-infected macrophages. Beta tubulin was used as loading control. Values are expressed as mean±SE, n=3.

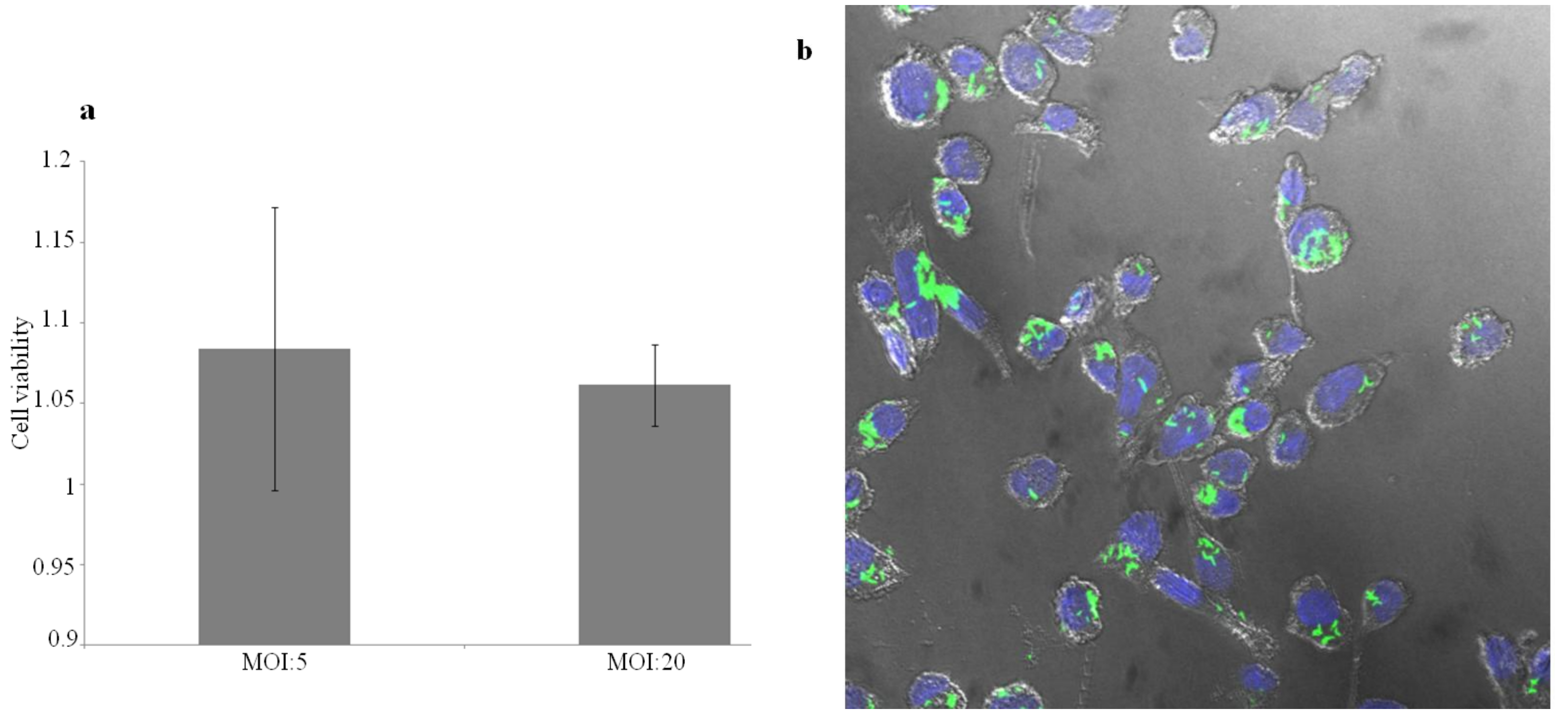

Supplementary figure S3. Cell viability is not affected at MOI:20 a) Viability of H37Rv-infected macrophages after 24h of infection at both MOI:5 and MOI:20 as calculated from MTT assay. Values represent the ratio of normalized OD of infected macrophages to uninfected macrophages. Values are expressed as mean±SE, n=6; b) macrophages infected with H37Rv-GFP at MOI:20, after 24h of infection showing intact nuclei (blue) and healthy morphology.

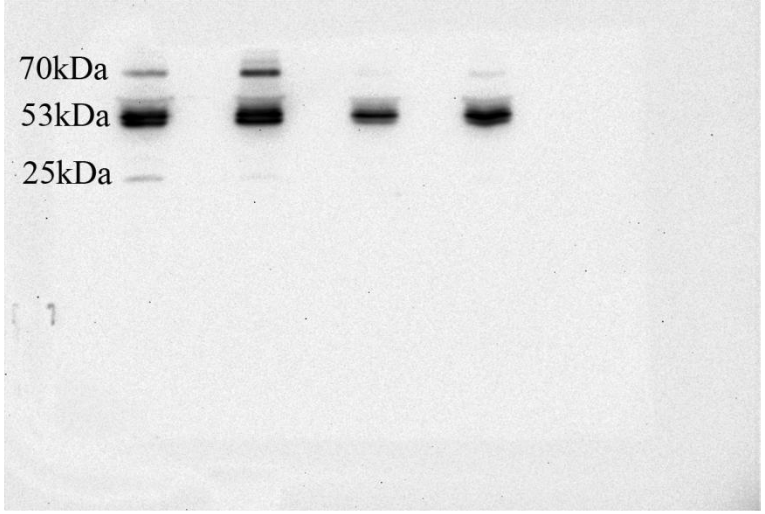

Supplementary figure S4. Full blot of Fig. 1c showing different bands of vimentin. PAGE was run in a 15% gel.

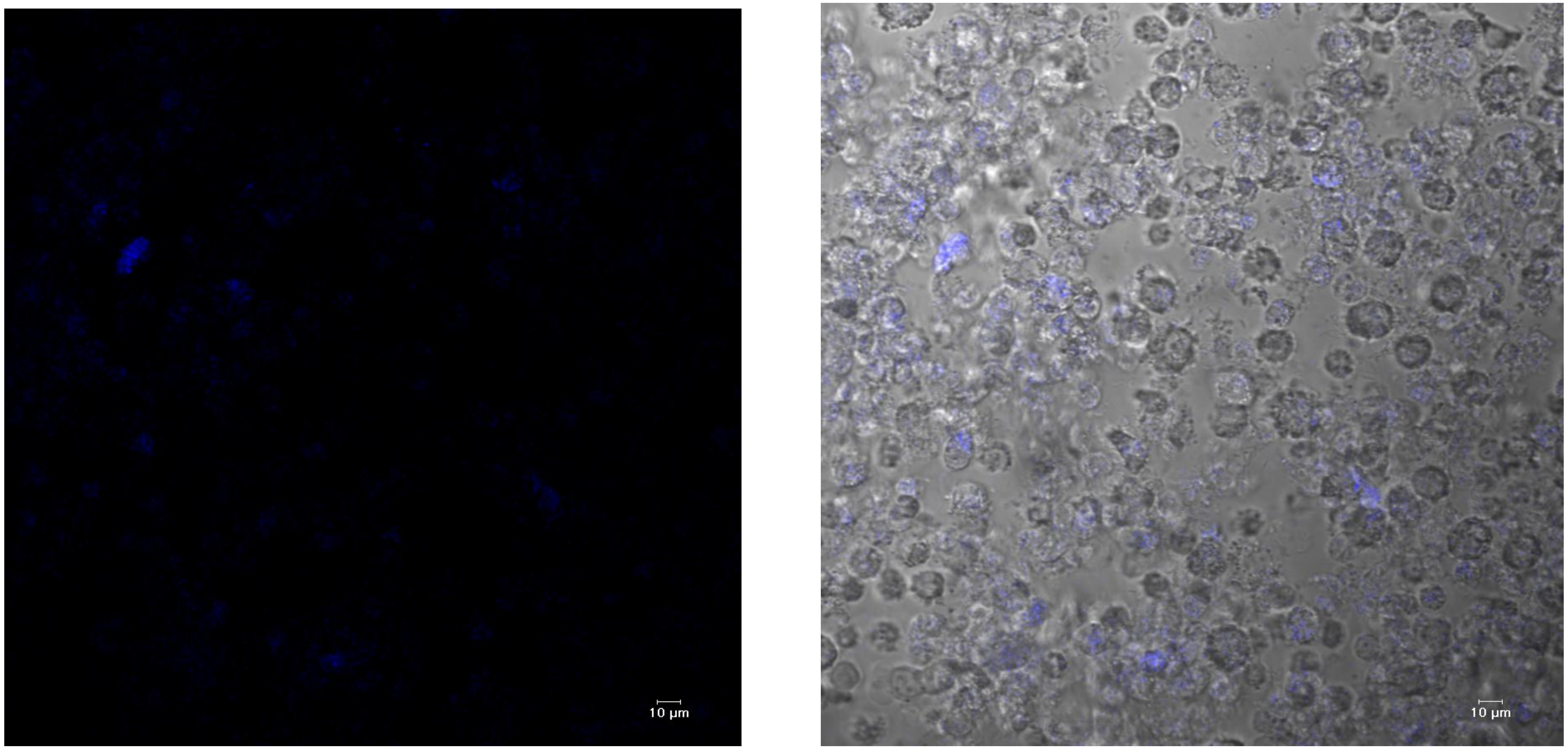

Imaged with 405nm laser Merged

Supplimentary figure S5. ESAT-6 expressing macrophages shown with ECFP reporter (blue)

Supplementary method

MTT assay: MTT reagent (M2128, Sigma) was dissolved in PBS at 5mg/ml. 20µl of MTT solution was added to 200µl of culture medium of infected macrophages in each well of a 96 well plate and incubated for 4h. After the incubation the solution was aspirated and 100µl of DMSO was added to each well. The content was mixed by keeping the plate on a rocker for 5 min and absorbance was measured at 570 nm. Background absorbance was measured at 690 nm and subtracted from the value at 570 nm.
